# Left Ventricular Myocardial Deformation Abnormalities in Ankylosing Spondylitis (Radiographic Axial Spondyloarthritis): Association With Disease Severity Assessed by Two-Dimensional Speckle-Tracking Echocardiography

**DOI:** 10.14740/cr2308

**Published:** 2026-10-12

**Authors:** Muhammad Saad Reihan, Mohammed Moanes Mohammed Mohyeldin, Ahmed Al-Habbaa, Mohamed Sayed Bashandy, Husna Irfan Thalib, Ibrahim Fawzy Ezz, Mohamed Al-Adl Ali Ali, Abdelwahab Nasrat Bayoumy, Mohamad Abdel-Moez Ali

**Affiliations:** a Cardiology Department, Faculty of Medicine, Al-Azhar University, New Damietta, Egypt; b Department of Internal Medicine, General Medicine Practice Program, Batterjee Medical College, Jeddah, Saudi Arabia; c Cardiology Department, Faculty of Medicine, Al-Azhar University, Cairo, Egypt; d Hayat National Hospitals, Jazan, Saudi Arabia; e General Medicine Practice Program, Batterjee Medical College, Jeddah, Saudi Arabia; f Department of Rheumatology and Rehabilitation, Al-Azhar University, Faculty of Medicine, Cairo, Egypt

**Keywords:** Ankylosing spondylitis, Axial spondyloarthritis, Radiographic axial spondyloarthritis, Speckle-tracking echocardiography, Global longitudinal strain, Left ventricular dysfunction, Myocardial deformation, Cardiovascular involvement, Disease severity, Sacroiliitis

## Abstract

**Background:**

Ankylosing spondylitis (AS), also termed radiographic axial spondyloarthritis (r-axSpA) under current Assessment of SpondyloArthritis international Society (ASAS) nomenclature, is a chronic inflammatory disorder associated with an increased risk of cardiovascular involvement, including subclinical myocardial dysfunction. Conventional echocardiography often fails to detect early myocardial abnormalities despite preserved left ventricular systolic function. Two-dimensional speckle-tracking echocardiography (2D-STE) enables sensitive assessment of myocardial deformation through global longitudinal strain (GLS), facilitating the early detection of subclinical cardiac involvement. This study aimed to evaluate left ventricular myocardial deformation in patients with AS using 2D-STE and investigate its relationship with disease severity and duration.

**Methods:**

This cross-sectional observational study included 35 patients with AS diagnosed according to the Modified New York criteria. All participants underwent comprehensive clinical evaluation, laboratory investigations, conventional transthoracic echocardiography, and 2D-STE. Disease activity was assessed using the Bath Ankylosing Spondylitis Disease Activity Index (BASDAI) and Ankylosing Spondylitis Disease Activity Score (ASDAS). Radiographic disease severity was determined using the modified New York sacroiliitis grading system. Correlations between myocardial deformation parameters, radiographic severity, and disease duration were analyzed using Pearson’s correlation coefficient.

**Results:**

The study population had a mean age of 33.9 ± 7.3 years, and 68.6% were male. Conventional echocardiography demonstrated preserved left ventricular systolic function, with a mean left ventricular ejection fraction of 65.6±5.0%. The mean GLS was −17.92±4.80%. Radiographic sacroiliitis grade demonstrated a significant positive correlation with global longitudinal strain (r = 0.345, P = 0.046), apical two-chamber longitudinal strain (r = 0.455, P = 0.008), and apical four-chamber longitudinal strain (r = 0.392, P = 0.022), whereas no significant association was observed with apical three-chamber strain, aortic root diameter, or valvular abnormalities. Disease duration showed a moderate negative correlation with GLS (r = −0.544, P = 0.001), indicating greater impairment of myocardial deformation in patients with longer-standing disease.

**Conclusions:**

Patients with AS demonstrated evidence of subclinical left ventricular myocardial dysfunction despite preserved conventional systolic function. Greater radiographic disease severity and longer disease duration were significantly associated with impaired myocardial deformation assessed by speckle-tracking echocardiography. These findings support the potential role of GLS as a sensitive imaging biomarker for the early detection of cardiac involvement in AS and highlight the importance of cardiovascular surveillance in patients with advanced or longstanding disease.

## Introduction

Ankylosing spondylitis (AS) is a chronic inflammatory rheumatic disease belonging to the spectrum of axial spondyloarthritis (axSpA), characterized primarily by inflammation of the sacroiliac joints and spine, leading to progressive structural damage, reduced spinal mobility, and impaired physical function [1]. In accordance with current ASAS nomenclature, patients fulfilling the modified New York radiographic criteria for AS, as applied in the present cohort, are classified as having radiographic axial spondyloarthritis (r-axSpA); the terms AS and r-axSpA are therefore used interchangeably throughout this manuscript, with r-axSpA highlighted at key points for consistency with updated terminology. Although musculoskeletal manifestations represent the hallmark of the disease, AS is increasingly recognized as a systemic inflammatory disorder with a wide range of extra-articular manifestations involving the cardiovascular, pulmonary, renal, gastrointestinal, and ocular systems. Cardiovascular involvement is among the most clinically significant extra-articular manifestations because it substantially contributes to long-term morbidity and premature mortality in affected individuals [2, 3].

Patients with AS exhibit a higher prevalence of cardiovascular disease compared with the general population, with epidemiological studies demonstrating an increased risk of ischemic heart disease, heart failure, cerebrovascular events, conduction abnormalities, and valvular heart disease [4]. Chronic systemic inflammation, endothelial dysfunction, accelerated atherosclerosis, oxidative stress, and immune-mediated myocardial injury have all been implicated in the pathogenesis of cardiovascular involvement in AS. Persistent inflammatory activity, particularly mediated through tumor necrosis factor-alpha (TNF-α), interleukin (IL)-17, and IL-23 signaling pathways, promotes myocardial remodeling and interstitial fibrosis, ultimately impairing ventricular function even before overt clinical manifestations become apparent [5, 6].

Cardiac abnormalities in AS have traditionally been described as involving the aortic root, aortic valve, mitral valve, and cardiac conduction system. Aortic root dilatation and aortic regurgitation result from chronic inflammation and fibrosis extending from the aortic annulus into the ascending aorta, whereas conduction disturbances arise from inflammatory infiltration and fibrosis of the atrioventricular node and interventricular septum [7, 8]. However, accumulating evidence suggests that myocardial involvement may occur independently of these classical manifestations and may remain clinically silent for many years. Histopathological studies have demonstrated diffuse myocardial fibrosis and inflammatory infiltration, providing a potential explanation for subclinical ventricular dysfunction observed in otherwise asymptomatic patients [9].

Conventional transthoracic echocardiography remains the first-line imaging modality for assessing cardiac structure and systolic function in patients with AS. Nevertheless, traditional parameters such as left ventricular ejection fraction (LVEF) often remain within normal limits until advanced myocardial impairment develops, limiting their sensitivity for detecting early myocardial dysfunction. Consequently, there has been increasing interest in advanced echocardiographic techniques capable of identifying subtle abnormalities before irreversible structural damage occurs [10].

Two-dimensional speckle-tracking echocardiography (2D-STE) has emerged as a highly sensitive and reproducible imaging technique for the assessment of myocardial deformation. Unlike conventional echocardiographic indices, speckle-tracking directly quantifies myocardial strain by tracking natural acoustic markers throughout the cardiac cycle, enabling objective evaluation of regional and global myocardial mechanics [11]. Global longitudinal strain (GLS) has become one of the most robust markers of subclinical left ventricular systolic dysfunction and has demonstrated superior sensitivity compared with LVEF in a variety of cardiovascular and systemic inflammatory diseases. Reductions in GLS may precede detectable changes in ejection fraction by several years, allowing earlier identification of myocardial involvement and facilitating timely therapeutic intervention [12].

Several investigators have reported impaired left ventricular longitudinal strain in patients with AS despite preserved conventional systolic function, suggesting the presence of occult myocardial dysfunction. Proposed mechanisms include chronic inflammatory injury, myocardial edema, microvascular dysfunction, diffuse interstitial fibrosis, and increased ventricular stiffness resulting from prolonged inflammatory activity [13]. Furthermore, disease duration and inflammatory burden have been suggested as important determinants of myocardial deformation abnormalities. Clinical indices such as the Ankylosing Spondylitis Disease Activity Score (ASDAS) and Bath Ankylosing Spondylitis Disease Activity Index (BASDAI), together with inflammatory biomarkers including erythrocyte sedimentation rate (ESR) and C-reactive protein (CRP), may therefore provide insight into the relationship between disease activity and cardiac involvement [14]. However, published studies have yielded inconsistent findings regarding the association between disease severity and myocardial strain, likely owing to differences in study populations, imaging protocols, disease duration, treatment exposure, and sample size.

Radiographic structural damage, reflected by the severity of sacroiliitis on conventional radiographs, represents cumulative disease burden and may serve as an indirect marker of chronic inflammation. Whether radiographic progression correlates with myocardial deformation abnormalities remains insufficiently investigated [15]. Likewise, the influence of disease duration on myocardial strain has not been consistently established, highlighting the need for further studies evaluating these associations using contemporary echocardiographic techniques [2, 15].

Given the growing recognition of cardiovascular disease as a major determinant of prognosis in patients with AS, early identification of subclinical myocardial dysfunction has important clinical implications. Detection of impaired myocardial deformation before the onset of overt heart failure or significant reductions in ejection fraction may permit closer cardiovascular surveillance, optimization of anti-inflammatory therapy, and implementation of preventive strategies aimed at reducing long-term cardiovascular complications [4, 5].

Therefore, the present study aimed to evaluate left ventricular myocardial deformation using 2D-STE in patients with AS and to investigate its relationship with disease severity, radiographic sacroiliitis grade, disease duration, and conventional echocardiographic findings. We hypothesized that greater disease severity and longer disease duration would be associated with impaired myocardial deformation despite preserved conventional measures of left ventricular systolic function.

## Materials and Methods

### Study design and participants

This cross-sectional observational study was conducted using data obtained from the medical records of patients attending the Cardiology and Rheumatology Departments at Al-Husain Hospital, Cairo, and New Damietta Hospital, Damietta, Egypt to evaluate left ventricular myocardial deformation in patients with AS using 2D-STE and to investigate its relationship with disease severity. Adult patients aged 18 years or older with a confirmed diagnosis of AS according to the modified New York criteria were consecutively recruited from the rheumatology outpatient clinic. Patients with known ischemic heart disease, heart failure, congenital heart disease, cardiomyopathy, uncontrolled hypertension, diabetes mellitus with established cardiovascular complications, chronic kidney disease, atrial fibrillation, significant valvular heart disease, poor echocardiographic image quality, or any other systemic disorder known to affect myocardial function were excluded. Patients with incomplete clinical or echocardiographic data were also excluded from the study. A total of 35 eligible patients were included in the final analysis. The corresponding patient flow, including the inclusion and exclusion criteria applied during recruitment, is summarized in Figure 1.

**Figure 1:**
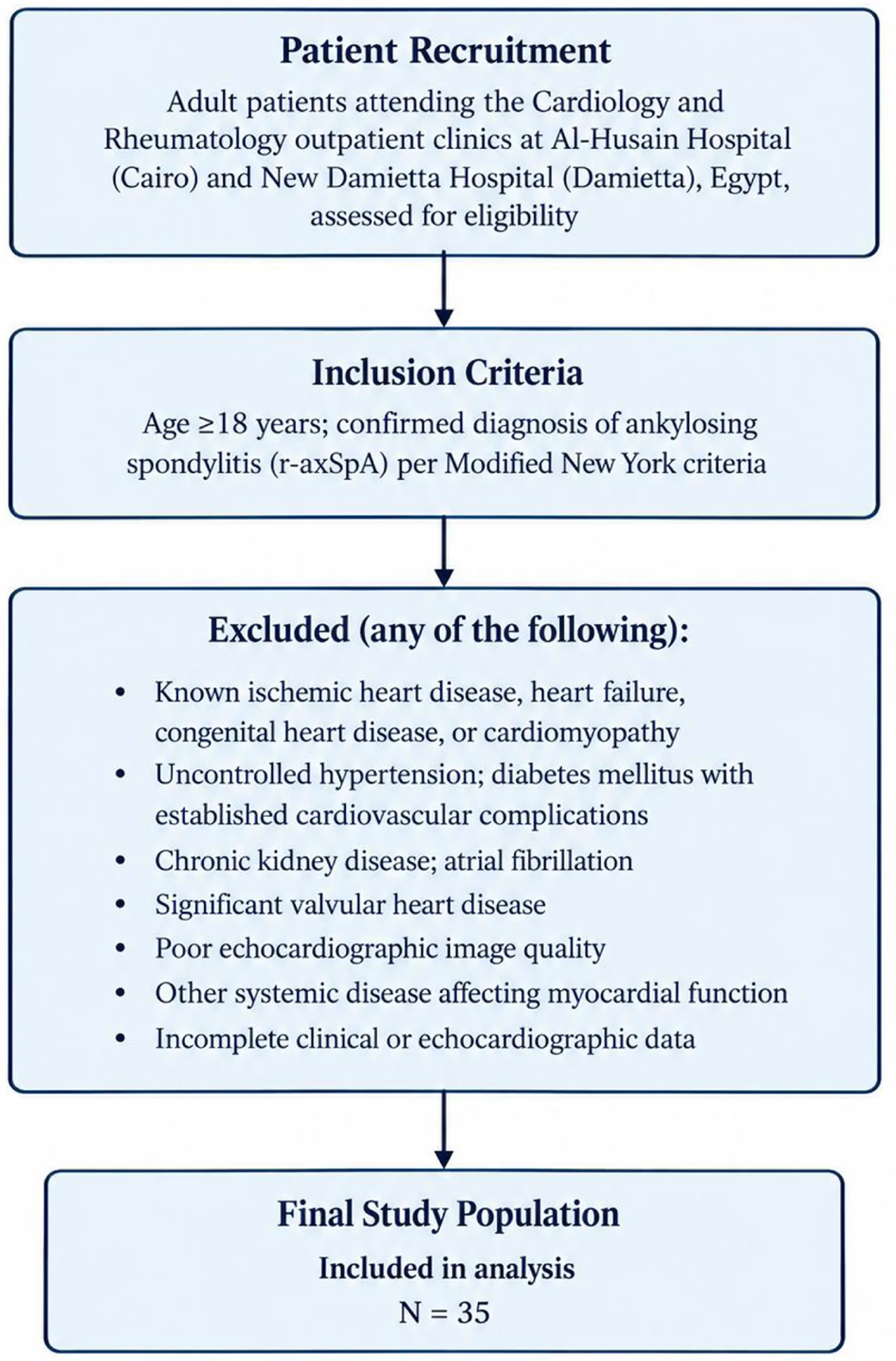
Flow diagram of the patient selection process, showing the eligibility criteria and exclusion categories applied at Al-Husain Hospital (Cairo) and New Damietta Hospital (Damietta), Egypt, and the derivation of the final study population (N = 35).

### Ethical considerations

The study protocol was approved by the Institutional Review Board of Al Azhar Faculty of Medicine (Approval No. 0000076) before participant recruitment. All procedures were conducted in accordance with the ethical principles outlined in the Declaration of Helsinki. Written informed consent was obtained from every participant before enrollment.

### Clinical and laboratory assessment

Demographic characteristics, including age, sex, body weight, height, and body mass index (BMI), were recorded for all participants. Clinical information including smoking status, family history of AS, history of peripheral arthritis, enthesitis, spinal pain, chest pain, uveitis, previous fractures, disease duration, and treatment duration was obtained through patient interviews and review of medical records.

Data on concurrent pharmacologic therapy, including non-steroidal anti-inflammatory drugs, conventional and biologic disease-modifying antirheumatic drugs, and cardiovascular medications such as angiotensin-converting enzyme inhibitors and angiotensin receptor blockers, were not systematically recorded in this retrospective cohort and could therefore not be analyzed as potential confounders or effect modifiers of myocardial strain; this constraint is acknowledged as a study limitation.

Disease activity was evaluated using the BASDAI together with the Ankylosing Spondylitis Disease Activity Score calculated using both erythrocyte sedimentation rate (ASDAS-ESR) and C-reactive protein (ASDAS-CRP). Laboratory investigations included ESR, CRP, and human leukocyte antigen-B27 (HLA-B27) status. Standard anteroposterior pelvic radiographs were obtained for all participants, and sacroiliac joint involvement was graded according to the modified New York radiographic grading system. Radiographic sacroiliitis grade was used as an indicator of cumulative structural disease severity.

### Echocardiographic assessment

All participants underwent comprehensive transthoracic echocardiography using a Philips iE33 ultrasound system (Philips Healthcare, Andover, MA, USA) equipped with an S5-1 sector array transducer (frequency range 1–5 MHz). Standard 2D and Doppler optimization settings were applied according to the manufacturer’s recommendations. Examinations were performed with the participants in the left lateral decubitus position by an experienced cardiologist who was blinded to the clinical and laboratory findings. Conventional two-dimensional, M-mode, pulsed-wave Doppler, color Doppler, and tissue Doppler imaging were acquired in accordance with the recommendations of the American Society of Echocardiography and the European Association of Cardiovascular Imaging.

Measurements included aortic root diameter, left atrial diameter, right ventricular diameter, interventricular septal thickness, left ventricular end-diastolic diameter, left ventricular end-systolic diameter, left ventricular posterior wall thickness, LVEF, left ventricular fractional shortening (LVFS), transmitral early (E) and late (A) diastolic velocities, E/A ratio, early diastolic mitral annular velocity (e′), E/e′ ratio, and indexed left atrial volume. Cardiac valves were systematically evaluated for the presence of calcification, stenosis, or regurgitation using two-dimensional imaging and Doppler techniques.

### Speckle-tracking echocardiography

Assessment of left ventricular myocardial deformation was performed using 2D-STE. Standard grayscale images were acquired from the apical four-chamber, two-chamber, and three-chamber views during breath-holding while maintaining a stable electrocardiographic recording. Images were obtained at an adequate frame rate and stored digitally for subsequent offline analysis using speckle tracking analysis software on the Philips iE33 echocardiography system powered by Philips QLAB Quantification Software.

For strain analysis, the left ventricular endocardial border was manually traced at end-systole, after which the software automatically generated the region of interest and tracked myocardial speckles throughout the cardiac cycle. Segments demonstrating inadequate tracking quality were manually adjusted or excluded from analysis. Peak systolic longitudinal strain was calculated separately for the apical four-chamber (Ap4-LS), apical two-chamber (Ap2-LS), and apical three-chamber (Ap3-LS) views. GLS was subsequently calculated as the average peak longitudinal strain derived from all analyzable left ventricular segments. Less negative GLS values were considered indicative of impaired myocardial systolic deformation despite preserved conventional systolic function.

### Outcome measures

The primary outcome of the study was GLS, which served as a marker of subclinical left ventricular systolic dysfunction. Secondary outcomes included regional longitudinal strain measurements obtained from the apical four-chamber, two-chamber, and three-chamber views, conventional echocardiographic parameters, valvular abnormalities, and the relationship between myocardial deformation parameters and disease duration, disease activity, and radiographic severity.

### Statistical analysis

Statistical analysis was performed using IBM SPSS Statistics version 26 (IBM Corp., Armonk, NY, USA). Continuous variables were assessed for normality using the Shapiro–Wilk test and are presented as mean ± standard deviation (SD). Categorical variables are expressed as frequencies and percentages. Correlations between radiographic sacroiliitis grade, disease duration, and echocardiographic parameters were evaluated using Pearson’s correlation coefficient. Correlation coefficients were interpreted according to conventional definitions, with values of 0.20–0.39 indicating weak correlations, 0.40–0.59 indicating moderate correlations, and values ≥ 0.60 indicating strong correlations. All statistical tests were two-tailed, and a P value of less than 0.05 was considered statistically significant.

## Results

### Patient characteristics

A total of 35 patients with AS (mean age 33.9 ± 7.3 years; 68.6% male) were included in the study. The cohort had a mean disease duration of 12.2 ± 6.1 years and demonstrated high overall disease activity (mean BASDAI 5.50 ± 1.23; mean ASDAS-CRP 4.07 ± 5.70). Full demographic, laboratory, and clinical characteristics including body habitus, inflammatory markers, HLA-B27 status, smoking status, comorbidities, family history, clinical manifestations, and radiographic sacroiliitis grade distribution are summarized in Table 1.

**Table 1:** Demographic and Clinical Characteristics of the Study Population (N = 35)

Variable	Value
Age (years), mean ± SD	33.9 ± 7.3
Male sex, n (%)	24 (68.6)
Female sex, n (%)	11 (31.4)
Weight (kg), mean ± SD	79.2 ± 13.9
Height (cm), mean ± SD	168.0 ± 29.6
BMI (kg/m^2^), mean ± SD	26.7 ± 4.5
Disease duration (years), mean ± SD	12.2 ± 6.1
Duration of treatment (years), mean ± SD	3.1 ± 1.1
ESR (mm/h), mean ± SD	45.2 ± 23.4
CRP (mg/L), mean ± SD	16.4 ± 11.9
HLA-B27 positive, n (%)	23 (65.7)
ASDAS-CRP, mean ± SD	4.07 ± 5.70
ASDAS-ESR, mean ± SD	3.41 ± 0.65
BASDAI, mean ± SD	5.50 ± 1.23
Current smokers, n (%)	8 (22.9)
Hypertension, n (%)	2 (5.7)
History of fracture, n (%)	6 (17.1)
Positive family history of AS, n (%)	17 (48.6)
Peripheral arthritis, n (%)	30 (85.7)
Spinal pain, n (%)	33 (94.3)
Enthesitis, n (%)	29 (82.9)
Chest pain, n (%)	27 (77.1)
Uveitis, n (%)	3 (8.6)

AS: ankylosing spondylitis; ASDAS: Ankylosing Spondylitis Disease Activity Score; BASDAI: Bath Ankylosing Spondylitis Disease Activity Index; BMI: body mass index; CRP: C-reactive protein; ESR: erythrocyte sedimentation rate; HLA-B27: human leukocyte antigen B27; SD: standard deviation.

### Radiographic severity and valvular abnormalities

Radiographic assessment demonstrated that most patients had moderate structural disease, with 48.6% exhibiting grade II sacroiliitis and 40.0% exhibiting grade III sacroiliitis, while only 11.4% had grade IV disease (Table 2).

**Table 2:** Radiographic Severity and Valvular Abnormalities

Sacroiliitis grade	n (%)
Grade II	17 (48.6)
Grade III	14 (40.0)
Grade IV	4 (11.4)
Valvular abnormalities	n (%)
No valvular abnormality	25 (71.4)
Mild MR	2 (5.7)
Mild AR	1 (2.9)
Calcified mitral and aortic valves	4 (11.4)
Calcific aortic valve with mild AR	1 (2.9)
Calcified mitral valve with mild MR and calcified aortic valve	2 (5.7)

AR: aortic regurgitation; MR: mitral regurgitation.

Echocardiographic evaluation showed that the majority of patients (71.4%) had no detectable valvular abnormalities. Among those with valvular involvement, calcific valvular changes were the most frequently observed abnormalities, whereas isolated mild mitral regurgitation and mild aortic regurgitation were less common (Table 2). Importantly, all valvular abnormalities identified in this cohort were mild in severity, with no evidence of hemodynamically significant regurgitation, valve stenosis, chamber enlargement, or measurable impairment of left ventricular systolic function, indicating that these findings were of limited clinical significance in the present population.

### Echocardiographic findings

Conventional transthoracic echocardiography demonstrated preserved cardiac chamber dimensions and left ventricular systolic function in the study population (Table 3). The mean LVEF was 65.6±5.0%, indicating preserved global systolic function. Left ventricular dimensions, including left ventricular end-diastolic diameter (LVEDD) and left ventricular end-systolic diameter (LVESD), as well as interventricular septal and posterior wall thicknesses, were within expected ranges. Similarly, measurements of the aortic root, left atrial diameter, and right ventricular diameter showed no marked structural abnormalities.

**Table 3:** Conventional Echocardiographic Parameters

Variable	Mean ± SD
Aortic root diameter (mm)	30.9 ± 3.8
Left atrial diameter (mm)	33.8 ± 5.5
Right ventricular diameter (mm)	21.6 ± 4.3
IVS thickness (mm)	8.2 ± 1.2
LVEDD (mm)	49.3 ± 3.3
LVPW thickness (mm)	8.0 ± 1.2
LVESD (mm)	31.8 ± 2.8
LVEF (%)	65.6 ± 5.0
LVFS (%)^a^	35.5 ± 4.1
Mitral E velocity (cm/s)^b^	74.7 ± 16.5
Mitral A velocity (cm/s)^b^	57.2 ± 14.2
E/A ratio‡	1.32 ± 0.28
Left atrial volume index (mL/m^2^)^a^	25.4 ± 10.5
Septal e′ velocity (cm/s)^c^	10.7 ± 2.9

^a^Available for 20 patients. ^b^Available for 33 patients. ^c^Available for 28 patients. IVS: interventricular septum; LVEDD: left ventricular end-diastolic diameter; LVPW: left ventricular posterior wall; LVESD: left ventricular end-systolic diameter; LVEF: left ventricular ejection fraction; LVFS: left ventricular fractional shortening; SD: standard deviation.

Assessment of diastolic function revealed a mean transmitral E/A ratio of 1.32 ± 0.28, with a mean septal e′ velocity of 10.7 ± 2.9 cm/s and a mean indexed left atrial volume of 25.4 ± 10.5 mL/m^2^, suggesting generally preserved diastolic function among the evaluated patients (Table 3).

### Left ventricular myocardial deformation

Speckle-tracking echocardiography demonstrated a mean GLS of −17.92±4.80%. The mean longitudinal strain values obtained from the apical four-chamber, two-chamber, and three-chamber views were −18.58±4.89%, −17.83±6.23%, and −16.66±4.91%, respectively. The mean E/e′ ratio was 7.42 ± 2.48, indicating normal left ventricular filling pressures in the subgroup of patients in whom this parameter was available (Table 4).

**Table 4:** Left Ventricular Myocardial Deformation Parameters

Parameter	Mean ± SD
E/e′ ratio^a^	7.42 ± 2.48
Apical four-chamber longitudinal strain (%)	−18.58 ± 4.89
Apical two-chamber longitudinal strain (%)	−17.83 ± 6.23
Apical three-chamber longitudinal strain (%)	−16.66 ± 4.91
Global longitudinal strain (GLS) (%)	−17.92 ± 4.80

^a^Available for 19 patients. SD: standard deviation.

### Correlation between disease severity and echocardiographic parameters

Correlation analyses demonstrated no significant association between radiographic sacroiliitis grade and the presence of valvular abnormalities (r = −0.188, P = 0.280) or aortic root diameter (r = 0.266, P = 0.123) (Table 5). Conversely, radiographic sacroiliitis grade showed a significant positive correlation with GLS (r = 0.345, P = 0.046), indicating progressively impaired myocardial deformation with increasing radiographic disease severity. Similar significant positive correlations were observed between sacroiliitis grade and apical two-chamber longitudinal strain (r = 0.455, P = 0.008) and apical four-chamber longitudinal strain (r = 0.392, P = 0.022). In contrast, no significant relationship was identified between sacroiliitis grade and apical three-chamber longitudinal strain (r = 0.196, P = 0.268) (Table 5).

**Table 5:** Correlation Between Disease Severity and Echocardiographic Parameters

Variable	Correlation coefficient (r)	P value
Sacroiliitis grade vs. valvular abnormalities	−0.188	0.280
Sacroiliitis grade vs. aortic root diameter	0.266	0.123
Sacroiliitis grade vs. global longitudinal strain	0.345	0.046*
Sacroiliitis grade vs. apical three-chamber LS	0.196	0.268
Sacroiliitis grade vs. apical two-chamber LS	0.455	0.008*
Sacroiliitis grade vs. apical four-chamber LS	0.392	0.022*
Disease duration vs. global longitudinal strain	−0.544	0.001*

*Statistical significance (P < 0.05).

Disease duration demonstrated a moderate negative correlation with GLS (r = −0.544, P = 0.001), suggesting that longer disease duration was associated with greater impairment of left ventricular myocardial deformation (Table 5).

## Discussion

The present study evaluated left ventricular myocardial deformation using 2D-STE in patients with AS and explored its relationship with disease severity and duration. The principal findings of this study were that patients with AS demonstrated evidence of impaired myocardial deformation despite preserved conventional left ventricular systolic function. Furthermore, increasing radiographic disease severity was significantly associated with worsening GLS and regional longitudinal strain, while longer disease duration correlated with greater impairment in GLS. Conversely, radiographic severity showed no significant association with valvular abnormalities or aortic root diameter [4, 5, 12–15].

Cardiovascular involvement represents one of the most important extra-articular manifestations of AS and has emerged as a major contributor to morbidity and mortality among affected patients. Although inflammatory changes involving the aortic root, cardiac valves, and conduction system have long been recognized, increasing evidence suggests that myocardial involvement may occur much earlier than clinically apparent cardiovascular disease. Because these myocardial abnormalities are frequently asymptomatic, conventional clinical assessment and routine echocardiography may fail to detect early dysfunction, highlighting the importance of more sensitive imaging techniques such as 2D-STE [4, 5].

In the present study, conventional echocardiographic parameters remained largely within normal limits. LVEF averaged 65.6%, and ventricular dimensions, wall thicknesses, and chamber sizes were generally preserved. These findings indicate that most patients had no evidence of overt structural heart disease or clinically detectable systolic dysfunction. Similar observations have been reported in previous studies, where conventional echocardiographic indices remained normal despite evidence of impaired myocardial mechanics detected by speckle-tracking imaging. This discrepancy reflects the limited sensitivity of LVEF, which often remains preserved until substantial myocardial damage has already occurred. Consequently, reliance solely on conventional echocardiographic parameters may underestimate the true prevalence of cardiac involvement in patients with AS.

Assessment of myocardial deformation using speckle-tracking echocardiography revealed a mean GLS of −17.92%, suggesting subtle impairment of left ventricular systolic function despite preserved ejection fraction. Longitudinal myocardial fibers are predominantly located within the subendocardium, making them particularly susceptible to chronic inflammation, oxidative stress, microvascular dysfunction, and interstitial fibrosis. GLS has therefore emerged as one of the earliest indicators of myocardial dysfunction and has consistently demonstrated greater sensitivity than conventional systolic indices in detecting subclinical cardiac disease. The reduced GLS observed in our study supports the growing body of evidence indicating that occult myocardial dysfunction is common in patients with AS.

One of the most important findings of the present study was the significant relationship between radiographic disease severity and myocardial deformation. Increasing sacroiliitis grade was associated with progressively impaired GLS as well as apical two-chamber and apical four-chamber longitudinal strain. These findings suggest that cumulative structural disease burden is accompanied by progressive deterioration in myocardial mechanics. Radiographic sacroiliitis reflects longstanding inflammatory damage rather than current inflammatory activity alone; therefore, its association with myocardial strain may indicate the cumulative effects of persistent systemic inflammation on cardiac tissue over time.

Chronic inflammation plays a central role in the pathogenesis of myocardial dysfunction in AS. Persistent activation of pro-inflammatory cytokines, including TNF-α, IL-17, and IL-23, promotes endothelial dysfunction, oxidative stress, fibroblast activation, and extracellular matrix remodeling. These processes ultimately result in diffuse myocardial fibrosis, increased ventricular stiffness, and impaired myocardial contractility. Histopathological studies have demonstrated inflammatory cell infiltration and replacement fibrosis within the myocardium of patients with AS, even in the absence of clinically evident cardiovascular disease. Such microscopic changes may explain why myocardial deformation abnormalities become detectable before measurable reductions in ejection fraction occur [6, 8].

Interestingly, no significant relationship was identified between radiographic disease severity and aortic root diameter or valvular abnormalities. Classical cardiovascular manifestations of AS, particularly aortic root dilatation and aortic regurgitation, are generally considered late complications that develop after prolonged disease duration. The relatively young age of our study population and the preservation of conventional echocardiographic parameters may explain the low prevalence of clinically significant valvular disease observed in this cohort. Notably, the valvular abnormalities identified in our cohort were uniformly mild and did not affect ventricular filling, chamber dimensions, or systolic function, further supporting their limited clinical relevance in this relatively young population. These findings are complementary to recent evidence demonstrating increased arterial stiffness and early vascular damage in patients with AS independent of traditional cardiovascular risk factors [16], suggesting that inflammatory vascular remodeling and myocardial deformation may represent parallel, yet mechanistically distinct, manifestations of cardiovascular involvement in this disease. These findings further emphasize that myocardial deformation imaging may identify cardiac involvement at an earlier stage than conventional structural echocardiographic assessment.

Disease duration demonstrated a moderate negative correlation with GLS, indicating greater myocardial dysfunction among patients with longer-standing disease. This observation is biologically plausible because prolonged exposure to systemic inflammation increases cumulative myocardial injury and fibrosis. Recurrent inflammatory activity over several years may gradually impair myocardial contractility through progressive remodeling of the extracellular matrix, ultimately producing measurable reductions in longitudinal strain while conventional systolic function remains preserved. This finding highlights the cumulative impact of chronic inflammation on myocardial function and suggests that duration of disease may represent an important determinant of cardiovascular risk in AS [4, 5, 15].

Several previous investigations have similarly reported impaired myocardial strain in patients with AS despite preserved LVEF. Although differences in study populations, imaging protocols, disease activity, treatment exposure, and sample size have resulted in some variability across published studies, the overall body of evidence supports the concept of subclinical myocardial involvement in AS [17]. Cardiac strain assessed by 2D-STE has also been examined in the context of pharmacologic disease control in axSpA and related autoimmune rheumatic diseases; a recent prospective 1-year echocardiographic study found no significant decline in GLS among patients with axSpA, rheumatoid arthritis, or psoriatic arthritis treated with Janus kinase inhibitors, suggesting that effective pharmacologic control of systemic inflammation may help preserve myocardial mechanics over time [18]. Our findings further contribute to this growing literature by demonstrating that myocardial deformation abnormalities are associated not only with disease duration but also with radiographic severity, suggesting that cumulative structural disease burden may be an important determinant of cardiac involvement.

An important consideration in interpreting these findings relates to the extent to which myocardial deformation reflects cumulative structural damage versus ongoing inflammatory activity. In the present study, radiographic sacroiliitis grade served as our principal marker of structural disease burden, whereas the relationship between GLS and disease activity indices (BASDAI, ASDAS) or inflammatory markers was not the focus of the primary analysis, precluding a formal comparison between damage-related and activity-related contributions to myocardial impairment. This distinction is clinically relevant, since the concept of difficult-to-manage, treatment-refractory axSpA, as recently defined through an ASAS consensus process, specifically identifies patients with persistent inflammatory activity that may occur independently of accumulated radiographic damage [19]. Future studies incorporating these disease-activity and treatment-refractory constructs alongside myocardial strain parameters would help clarify whether cardiac involvement in axSpA is driven predominantly by ongoing inflammation, cumulative structural burden, or both, and could help identify patient subgroups who may benefit most from intensified cardiovascular surveillance.

The absence of significant correlations between radiographic severity and apical three-chamber longitudinal strain may reflect regional heterogeneity in myocardial deformation or may simply be attributable to the relatively small sample size. Regional strain measurements are generally more variable than GLS because they are influenced by image quality, segmental tracking accuracy, and physiological differences in myocardial contraction. Consequently, GLS remains the preferred and most reproducible parameter for evaluating global myocardial systolic performance [20].

From a clinical perspective, the findings of this study have important implications. Cardiovascular disease remains a major cause of morbidity among patients with AS, yet myocardial involvement frequently remains clinically silent until advanced stages. Incorporating speckle-tracking echocardiography into cardiovascular assessment, particularly for patients with longstanding disease or advanced radiographic sacroiliitis, may facilitate earlier identification of subclinical myocardial dysfunction. Early recognition of impaired myocardial deformation may allow closer cardiovascular surveillance, optimization of anti-inflammatory therapy, and timely implementation of preventive strategies aimed at reducing future cardiovascular complications.

The present study has several strengths. It utilized 2D-STE, one of the most sensitive non-invasive techniques currently available for assessing myocardial mechanics. In addition, the study evaluated the relationship between myocardial deformation and multiple indicators of disease burden, including radiographic severity and disease duration, thereby providing a comprehensive assessment of cardiovascular involvement in AS.

### Limitations

First, the relatively small sample size may have limited statistical power and reduced the ability to detect weaker associations.

Second, the cross-sectional design precludes establishing causal relationships between disease severity and myocardial dysfunction.

Third, the absence of a healthy control group is an important limitation that restricts direct comparison of myocardial strain values with individuals without AS; future studies incorporating age- and sex-matched healthy controls are needed to confirm the specificity of the observed strain abnormalities.

Fourth, inflammatory biomarkers were assessed at a single time point and may not accurately reflect cumulative inflammatory burden throughout the disease course.

Fifth, data on concurrent pharmacologic therapy including non-steroidal anti-inflammatory drugs, conventional and biologic disease-modifying antirheumatic drugs, and cardiovascular medications such as angiotensin-converting enzyme inhibitors and angiotensin receptor blockers were not systematically available in this retrospective cohort, precluding assessment of their potential influence on myocardial strain.

Finally, the study was conducted at a single center, which may limit the generalizability of the findings to broader patient populations.

Despite these limitations, the present study provides further evidence that subclinical myocardial dysfunction is present in patients with AS even when conventional echocardiographic parameters remain normal. The observed associations between myocardial deformation, radiographic severity, and disease duration suggest that chronic inflammatory burden contributes to progressive impairment of myocardial mechanics. Future prospective multicenter studies with larger sample sizes and longitudinal follow-up are warranted to determine whether early abnormalities in GLS predict future cardiovascular events and whether aggressive control of systemic inflammation can prevent or reverse myocardial dysfunction in patients with AS.

### Conclusion

The present study demonstrates that patients with AS (r-axSpA) exhibit evidence of subclinical left ventricular myocardial dysfunction despite preserved conventional systolic function. The 2D-STE identified impaired myocardial deformation that was not apparent on standard echocardiographic assessment, underscoring its superior sensitivity for detecting early cardiac involvement. Importantly, increasing radiographic sacroiliitis severity and longer disease duration were significantly associated with worsening GLS, suggesting that cumulative inflammatory burden contributes to progressive myocardial impairment. These findings highlight the potential value of GLS as a sensitive, non-invasive imaging biomarker for the early identification of cardiac involvement in AS. Routine cardiovascular evaluation incorporating speckle-tracking echocardiography, particularly in patients with longstanding disease or advanced radiographic changes, may facilitate earlier diagnosis of subclinical myocardial dysfunction and allow timely implementation of preventive and therapeutic strategies. Larger prospective multicenter studies with longitudinal follow-up are warranted to determine the prognostic significance of impaired myocardial deformation and to establish its role in cardiovascular risk stratification and disease monitoring in patients with AS.

## Data Availability

The datasets generated and analyzed during the current study are available from the corresponding author upon reasonable request.

## Abbreviations

AR: aortic regurgitation
AS: ankylosing spondylitis
axSpA: axial spondyloarthritis
r-axSpA: radiographic axial spondyloarthritis
ASDAS: Ankylosing Spondylitis Disease Activity Score
BASDAI: Bath Ankylosing Spondylitis Disease Activity Index
BMI: body mass index
CRP: C-reactive protein
ESR: erythrocyte sedimentation rate
GLS: global longitudinal strain
HLA-B27: human leukocyte antigen B27
IVS: interventricular septum
LA: left atrium
LVEDD: left ventricular end-diastolic diameter
LVEF: left ventricular ejection fraction
LVESD: left ventricular end-systolic diameter
LVFS: left ventricular fractional shortening
LVPW: left ventricular posterior wall
MR: mitral regurgitation
STE: speckle-tracking echocardiography
2D-STE: two-dimensional speckle-tracking echocardiography
